# Association between white matter lesions and cerebral Aβ burden

**DOI:** 10.1371/journal.pone.0204313

**Published:** 2018-09-24

**Authors:** Hyon-Ah Yi, Kyoung Sook Won, Hyuk Won Chang, Hae Won Kim

**Affiliations:** 1 Department of Neurology, Keimyung University Dongsan Medical Center, Daegu, Republic of Korea; 2 Department of Nuclear Medicine, Keimyung University Dongsan Medical Center, Daegu, Republic of Korea; 3 Department of Radiology, Semyung Radiology Clinic, Gumi, Republic of Korea; Nathan S Kline Institute, UNITED STATES

## Abstract

**Introduction:**

White matter lesions (WMLs), detected as hyperintensities on T2-weighted MRI, represent small vessel disease in the brain and are considered a potential risk factor for memory and cognitive impairment in older adults. The purpose of this study was to evaluate the association between WMLs and cerebral amyloid-β (Aβ) burden in patients with cognitive impairment.

**Methods:**

A total of 83 patients with cognitive impairment, who underwent brain MRI and F-18 florbetaben PET, were included prospectively: 19 patients were cognitively unimpaired, 30 exhibited mild cognitive impairment (MCI), and 34 exhibited dementia. The Fazekas scale was used to quantify WMLs on T2-weighted brain MR images. Cerebral Aβ burden was quantitatively estimated using volume-of-interest analysis. Differences in cerebral Aβ burden were evaluated between low-WML (Fazekas scale ≤1) and high-WML (Fazekas scale ≥2) groups. The relationship between the Fazekas rating and cerebral Aβ burden was evaluated using linear regression analysis after adjusting for age and sex.

**Results:**

In the overall cohort, the high-WML group exhibited significantly higher Aβ burden compared with the low-WML group (*P* = 0.011) and cerebral Aβ burden was positively correlated with Fazekas rating (*β* = 0.299, *P* = 0.006). In patients with MCI, the high-WML group exhibited significantly higher Aβ burden compared with the low-WML group (*P* = 0.019) and cerebral Aβ burden was positively correlated with Fazekas rating (*β* = 0.517, *P* = 0.003).

**Conclusion:**

The presence of WMLs was associated with cerebral Aβ burden in patients with MCI. Our findings suggest that small vessel disease in the brain is related to Alzheimer’s disease pathology.

## Introduction

White matter lesions (WMLs), which are typically detected as hyperintensities on T2-weighted magnetic resonance imaging (MRI), are common in the older adult population [1] and considered a potential risk factor for memory and cognitive impairment [2]. WMLs are associated with an increased incidence of dementia [3] and development of white matter microstructural changes during the presymptomatic stage of Alzheimer’s disease (AD) [4]. While the etiology and pathophysiology of WMLs are not completely understood, these lesions have been attributed to small vessel disease [5].

Epidemiological, biochemical, genetic, and animal studies of AD have suggested different origins for this complex disease process, leading to different theories regarding the etiology of AD. One theory is the amyloid-β (Aβ) cascade hypothesis, which proposes that deposition of the Aβ peptide in the brain is a central event in the pathogenesis of AD [6]. Along with Aβ, the microtubule-associated protein tau plays a key role in AD pathology by forming intracellular neurofibrillary tangles [7]. To date, a clear pathophysiology for AD exacting the contributions of each pathological protein has not been confirmed [8]. Recently, several studies have shown associations between WMLs and AD pathology [9–11]. A large population-based study has reported that WMLs contribute to brain atrophy patterns in regions associated with AD [12], and another study has proposed that the appearance of WMLs doubles the risk of AD [5]. One study using the second phase of the AD neuroimaging initiative (ADNI-2) cohort showed that WMLs affect the clinical and pathological processes of AD both directly and by interacting with tau [11]. Thus, if WMLs influence AD development, strategies to prevent small vessel disease may help to decrease the prevalence of AD or prevent its progression.

There is considerable indirect evidence that WMLs contribute to development of AD; however, there is insufficient direct evidence for the relationship between WMLs and AD pathology. Positron emission tomography (PET) with F-18 florbetaben (FBB) has shown potential for *in vivo* identification of cerebral Aβ pathology in patients undergoing assessment for AD [13]. Thus, we conducted the present study to evaluate the relationship between WMLs and cerebral Aβ burden in patients with cognitive impairment, using F-18 FBB PET.

## Material and methods

### Study population

This prospective study included a consecutive series of patients (age, 50–90 years), who visited the memory clinic at Keimyung University Dongsan Medical Center for the evaluation of cognitive function between June 2015 and January 2017. Standard clinical and neuropsychological evaluations were conducted. All patients were divided into three syndromal categories based on the 2018 National Institute on Aging-Alzheimer’s Association Research Framework: cognitively unimpaired (CU), mild cognitive impairment (MCI) due to AD, and dementia due to AD [14]. The Mini-Mental State Examination (MMSE), Digit Span Memory Test, Korean-Boston Naming Test (K-BNT), and Rey-Osterrieth Complex Figure Test and Recognition Trial (RCFT) were used to assess cognitive function [15]. All participants underwent brain MRI and F-18 FBB PET within 4 weeks of visiting the clinic. Patients with the following were excluded: an MMSE score <10; conditions that could affect cognition, including vascular dementia; a history of psychiatric episodes or substance abuse; or a previous diagnosis of dementia. Written informed consent was obtained from all participants or caregivers prior to enrollment in the study. The patient’s capacity to provide consent was determined by the MMSE score. In cases of participants with severely impaired cognitive function (MMSE <15), written informed consent was obtained from the caregivers. The legal caregivers were identified by a Family Relations Certificate. The institutional review board of Dongsan Medical Center approved this study, including the procedure in which caregivers provided consent on behalf of patients.

### Brain MRI

Brain MRI was performed using a 3T Signa Excite scanner (GE Healthcare, Milwaukee, WI, USA). For T1-weighted imaging, a high-resolution, three-dimensional spoiled gradient echo sequence was obtained (repetition time, 6 ms; echo time, 2.2 ms; 512 × 320; 152 axial slices; slice thickness, 5 mm; field of view, 210 mm). For T2-weighted images, a fast-spin echo sequence was obtained (repetition time, 4000 ms; echo time, 110 ms; 512 × 320; 152 axial slices; slice thickness, 5 mm; field of view, 210 mm).

The presence of WMLs was scored on axial T2-weighted MR images using the modified Fazekas scale [16]. Two researchers, blinded to the clinical information, independently rated periventricular and deep white matter hyperintensities. Periventricular white matter hyperintensity scores ranged from 0–3: 0, absence; 1, caps or pencil-thin lining; 2, smooth halo; and 3, irregular periventricular white matter hyperintensity extending into the deep white matter. The deep white matter hyperintensity scores ranged from 0–3: 0, absence; 1, punctuated foci; 2, beginning confluence of foci; and 3, large confluent areas. The Fazekas scale adopts the highest periventricular or deep white matter hyperintensity score. In the event of any disagreement between the two raters, a consensus was reached with the help of a neuroradiologist (H.W.C). Patients with a Fazekas scale score of 0 or 1 were classified into the low-WML group, and patients with a Fazekas scale score of 2 or 3 were classified into the high-WML group. Differences in cerebral Aβ burden were evaluated between the low- and high-WML groups.

### Amyloid PET

F-18 FBB positron emission tomography (PET) images were acquired using a PET/CT system (Biograph mCT-64, Siemens Healthcare, Knoxville, TN) in the three-dimensional mode. Images were acquired 90–100 min after intravenous injection of 300 MBq of F-18 FBB. A light, foam-rubber holder was applied for fixation of the head. PET images were reconstructed using an ordered-subset expectation maximum iterative reconstruction algorithm. Non-enhanced low-dose CT images were acquired for attenuation correction and localization.

Image processing was performed using SPM5 (Wellcome Department of Imaging Neuroscience, Institute of Neurology, University College London) in conjunction with MATLAB 2013a (MathWorks Inc., MA, USA). All reconstructed PET images were spatially normalized to Talairach space. PET images were analyzed quantitatively based on volumes of interest (VOIs) using the PMOD software program (PMOD Technologies Ltd, Zurich, Switzerland), as previously described [17]. Each MR and PET image was coregistered with a standard mutual information algorithm and spatially normalized. Following this, an automated anatomical labeling template was applied for standardized, regional brain VOI sampling of count densities. VOIs were individually defined on both hemispheres in the frontal, temporal, parietal, and occipital cortices; anterior and posterior cingulate cortices; and the cerebellar cortex. Standardized uptake values were obtained from these defined regional VOIs. Regional standardized uptake value ratios (SUVRs) were calculated by dividing the standardized uptake value for each regional VOI by the standardized uptake value for the cerebellar cortex as a reference region. A composite SUVR was calculated by dividing the mean of the standardized uptake values for the frontal, temporal, and parietal cortices, and the cingulate, by the standardized uptake value for the cerebellar cortex, as previously described [18]. The regional and composite SUVRs were used to evaluate the relationship between cerebral Aβ burden and WMLs. Patients with a composite SUVR of ≥1.39, which has previously been reported as a cut-off value that reflects an abnormally high cerebral Aβ burden, were considered positive for Aβ. Patients with a composite SUVR of <1.39 were considered negative for Aβ [18].

### Statistical analyses

All statistical analyses were performed using IBM SPSS statistics for Windows, V.20 (IBM Corp). Variables such as age, education, MMSE, Digit span, K-BNT, RCFT, and SUVR, which showed normal distribution, were compared between groups using one-way ANOVA. Bonferroni post-hoc analysis was used for between-group comparisons. The Fazekas rating, which was not normally distributed, was compared using the Kruskal Wallis test. Differences in sex, APOE4+, and Aβ positivity were compared between groups using Fisher’s exact test. Differences in regional and composite SUVR (cerebral Aβ burden) between low- and high-WML groups were evaluated using an independent two-sample *t*-test. Additionally, we assessed the relationship between the SUVR and Fazekas rating using Spearman’s correlation analysis and multiple linear regression analysis, adjusted for age and sex. We did not include the apolipoprotein E4 gene as a covariate in the primary model to avoid variance inflation, given its strong correlation with cerebral Aβ burden [19]. A *P* value of <0.05 was considered statistically significant. Data for all study variables are expressed as means ± standard deviation.

## Results

### Characteristics

**Fig 1:**
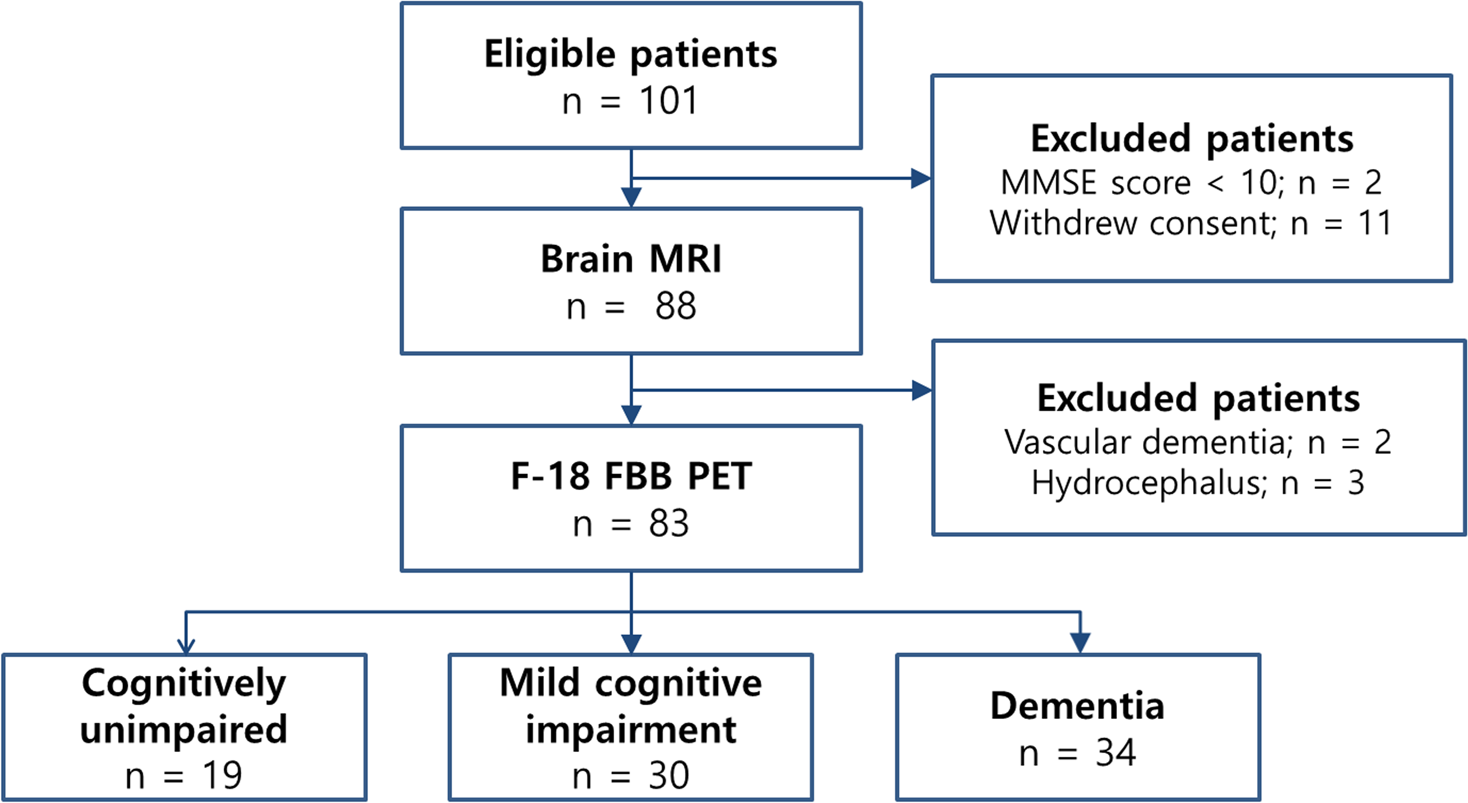
Flow diagram of the study population.

A total of 83 patients were enrolled in this study. Patients were divided into the following categories: CU (n = 19), MCI (n = 30), and dementia (n = 34). A flowchart of the study population is shown in Fig 1. We further classified the patients into low-WML (n = 51) and high-WML (n = 32) groups. The mean time interval between brain MRI and F-18 FBB PET was 7.4 ± 7.8 days (range, 0–27 days). Patient characteristics are described in Table 1.

**Table 1:** Patient characteristics*.

Characteristic	Overall (n = 83)	CU^†^ (n = 19)	MCI^‡^ (n = 30)	Dementia (n = 34)	*P value*	Post-hoc results
Age, y	65.7 (9.3)	62.5 (5.5)	64.9 (9.9)	68.2 (9.9)	0.083	―
Female, n	51	12	17	22	0.792	―
Education, y	11.5 (5.4)	14.2 (3.4)	12.8 (5.5)	8.8 (8.8)	0.061	―
APOE4+, n	25	4	7	14	0.326	―
MMSE	24.2 (5.2)	28.7 (1.4)	25.7 (2.5)	20.1 (5.4)	0.001	CN>MCI>AD
Digit span	9.3 (2.8)	11.7 (2.3)	10.0 (2.2)	7.5 (2.3)	<0.001	CN>MCI>AD
K-BNT^§^	42.7 (12.5)	51.8 (3.9)	46.3 (9.4)	34.4 (13.2)	<0.001	CN, MCI>AD
RCFT^‖^^,^	27.9 (8.6)	33 (2.4)	30.6 (5.3)	21.9 (10.3)	<0.001	CN, MCI>AD
Fazekas scale	1.0 (1.0)	0.5 (0.8)	0.8 (0.9)	1.4 (1.0)	0.001	CN, MCI<AD
Grade 1, n	38	14	16	8	―	―
Grade 2, n	13	1	5	7	―	―
Grade 3, n	28	4	9	15	―	―
Grade 4, n	4	0	0	4	―	―
Composite SUVR for Aβ	1.50 (0.25)	1.32 (0.10)	1.45 (0.22)	1.55 (0.31)	0.006	CN, MCI<AD
Aβ positivity, n	43	5	15	23	0.016	CN, MCI>AD

*Values represent means (SD)

^†^CU: cognitively unimpaired; ^‡^MCI: mild cognitive impairment

^§^K-BNT: Korean-Boston Naming Test

^‖^RCFT: Rey-Osterrieth Complex Figure Test and Recognition Trial

### WML and cerebral Aβ

In the overall cohort, the high-WML group exhibited significantly higher composite SUVRs compared with the low-WML group (*P* = 0.011; Table 2). In addition, the regional SUVRs in the frontal, temporal, parietal, and cingulate cortices in the high-WML group were significantly higher than those in the low-WML group (S1 Table). Spearman’s correlation analysis revealed a significant positive correlation between Fazekas rating and composite SUVR (*r* = 0.320, *P* = 0.003). Furthermore, linear regression analysis, adjusted for age and sex, showed that the Fazekas rating was associated with composite SUVR (*β* = 0.299, *P* = 0.006; Table 3), with a greater WML burden associated with a greater cerebral Aβ burden.

**Table 2:** Cerebral Aβ burden between low- and high-WML groups*.

Group	WML^†^ burden		*P value*
	Low	High	
Overall	1.41 (0.20)	1.55 (0.31)	0.011
CU^‡^	1.33 (0.13)	1.39 (0.22)	0.523
MCI^§^	1.36 (0.18)	1.59 (0.31)	0.019
Dementia	1.54 (0.22)	1.57 (0.33)	0.762

*All values are mean composite SUVR (SD)

^†^WML: white matter lesion

^‡^CU: cognitively unimpaired

^§^MCI: mild cognitive impairment

**Table 3:** Relationship between cerebral Aβ burden and Fazekas rating*.

Group	Adjusted *R*^2^	Standardized *β*	*P value*
Overall	0.079	0.300	0.006
CU^†^	―	0.244	0.105
MCI^‡^	0.242	0.517	0.003
Dementia	―	–0.035	0.342

*Values represent standardized linear regression coefficients (*β*) of the correlation between cerebral Aβ burden with Fazekas scale after controlling for age and sex in overall patients and each group

^†^CU: cognitively unimpaired

^‡^MCI: mild cognitive impairment

In patients with MCI, the high-WML group exhibited significantly higher composite SUVRs compared with the low-WML group (*P* = 0.019; Table 2). All regional SUVRs in the high-WML group, except for the right temporal cortex, were significantly higher than those in the low-WML group in patients with MCI (S1 Table). Spearman’s correlation analyses showed a significant positive correlation between Fazekas rating and composite SUVR in patients with MCI (*r* = 0.613, *P* < 0.001). Furthermore, multiple linear regression analysis, adjusted for age and sex, showed an association between Fazekas rating and composite SUVR in patients with MCI (*β* = 0.517, *P* = 0.003; Table 3). There were no significant differences in composite and regional SUVRs between the low- and high-WML groups in CU patients or patients with dementia. In addition, there was no significant correlation between Fazekas rating and composite SUVR in CU patients (*r* = 0.228, *P* = 0.348) or patients with dementia (*r* = –0.049, *P* = 0.784).

## Discussion

In the present study, we found a positive correlation between WML and cerebral Aβ burden, after adjusting for age and sex. This finding provides additional evidence that the presence of WMLs is associated with AD pathology. This finding is consistent with several studies that have reported an association between cerebrovascular disease and AD [20]. Previous data have indicated a considerable overlap between cerebrovascular disease and AD, with increased prevalence of WML; these lesions lower the threshold for clinical expression of dementia at a certain burden of AD pathology [21, 22]. Recently, vascular risk factors, including hypertension, smoking, and hypercholesterolemia, were revealed as clinical risk factors for the diagnosis of AD and presence of AD pathology [23–25]. Additionally, AD pathology, in the form of cerebral amyloidosis, can affect vascular and endothelial function, which may further impair vascular mechanisms and the elimination of cerebral Aβ [24].

WMLs are frequently observed as white matter hyperintensities on brain T2-weighted images in the older adults, particularly in patients with hypertension and stroke [3]. These lesions can occur as a consequence of ischemia and may be caused by small vessel disease [5]. However, recent studies have shown that WMLs can be caused by variable pathologies. One study used biochemical analysis to show that WMLs are associated with demyelination and degenerative axonal loss, but not ischemic damage, in patients with AD [26]. Another autopsy study has reported that WMLs are associated with cerebrovascular neuropathology (amyloid angiopathy, microinfarcts, infarcts, lacunes), and tau pathology [27]. Although the etiology and pathophysiology of WMLs are not fully understood, recent studies have added to the increasing evidence that WMLs are important in the development of AD. A previous study used immunohistochemistry to show a significant association between composite pathological score for AD at autopsy and WML score derived from T2-weighted images in a cohort of older adults [9]. In addition, a large population-based study found that WMLs contributed to brain atrophy patterns in regions associated to AD [12]. An MRI study has shown that the pattern of cerebral Aβ distribution is colocalized with default mode network activity prior to the clinical onset of AD [28]. Additionally, a study using amyloid PET and functional MRI showed an increase in WML burden and decreased functional connectivity of the prefrontal and temporal cortices in patients with MCI and AD, using information extracted from the ADNI database [10]. Furthermore, another study revealed that WML burden is associated with cerebral Aβ burden in cognitively normal subjects from the ADNI-2 cohort and Parkinson's progression markers initiative cohort [29], and a study with cerebrospinal fluid analysis showed that more pronounced WMLs were strongly and consistently associated with lower levels of Aβ-38 and Aβ-40 in 831 subjects with cognitive performance ranging from normal to AD [30]. A longitudinal study using ADNI data demonstrated that MCI patients who converted to AD showed faster WML burden progression compared with MCI patients who did not convert to AD [31]. In agreement with these previous studies, we found that the high-WML group exhibited a greater cerebral Aβ burden compared with the low-WML group in the overall cohort, and in patients with MCI. In addition, cerebral Aβ burden was positively correlated with WML burden in our overall cohort and in patients with MCI.

We found no significant correlation between WML burden and cerebral Aβ burden in patients with dementia in the present study. Previous prospective cohort studies have shown slow rates of cerebral Aβ accumulation in patients with MCI and a plateau of cerebral Aβ accumulation in patients with dementia [32]. In the present study, cerebral Aβ burden was higher in patients with dementia compared with CU patients and patients with MCI, and cerebral Aβ accumulation in patients with dementia was considered to have nearly reached a plateau [13]. This may have led to the discordant results in patients with dementia. Moreover, the present study revealed no significant difference in cerebral Aβ burden between the high- and low-WML groups in CU patients, in contrast to a previous study using ADNI data that revealed an association between the burden of WML and cerebral Aβ in CU patients [29]. This could be attributed to the small number of patients in the CU group in our study.

Some investigators have hypothesized that the primary event in the onset of AD is small vessel disease in the brain [33, 34]. According to this ischemic theory of AD, neuronal cells are damaged by microvascular ischemia, followed by impairment of the blood-brain barrier, which results in the release of neurotoxic Aβ peptides from the blood and subsequent infiltration into neuronal cells and the brain parenchyma [35]. In addition, the narrowing of small vessel lumens by Aβ plaques is a late effect of ischemia that leads to secondary ischemic brain episodes; this vicious cycle eventually leads to impairment of the neuronal network [33, 36]. Studies using rat models of ischemia support the ischemic theory of AD: they report increased levels of Aβ precursor proteins and the accumulation of endogenous Aβ peptides in the brain following transient cerebral artery occlusion [37, 38]. Another neuropathological study has shown the selective vulnerability of the hippocampus to ischemic insult in animal models. In accordance with this ischemic theory, our present study revealed an association between WMLs and cerebral Aβ burden, suggesting that small vessel disease, together with Aβ pathology, contributes to the development of AD. In addition to Aβ pathology, paired helical filaments of hyperphosphorylated tau protein are a major component of neurofibrillary tangles, which are a pathologic hallmark of AD [7]. In contrast to Aβ pathology, which does not reflect disease progression, cortical tau pathology correlates with the degree of cortical neuronal loss and clinical severity of dementia [39]. Further understanding of how WMLs influence AD pathophysiological progression calls for studies that investigate the relationship between WMLs and tau burden.

The present study has some limitations. WML burden was manually assessed using the Fazekas rating scale, which could have influenced our results by underestimating or overestimating WML burden. However, even with current quantitative methods to assess WML burden, lesion measurements and brain tissue parameters can be affected during image processing, differentiation from artifacts, and coregistration [40]. Furthermore, there is no standard/clinically-accepted method to quantitatively measure WML burden using T2-weighted MR images. The Fazekas rating scale is a simple and widely used system for evaluation of WMLs, and the consistency of this scoring system has been demonstrated [41]; therefore, our results should be minimally affected. Another limitation is that we could not assess changes in cerebral Aβ burden with the progression of WMLs, due to the cross-sectional design of this study. In contrast to the results of the present study, cerebral Aβ burden may affect development of WMLs [42]. Further longitudinal studies with larger sample sizes are necessary to evaluate the contribution of small vessel disease to cerebral Aβ accumulation.

## Conclusion

WMLs are associated with cerebral Aβ burden in patients with MCI. Our findings suggest that small vessel disease is related to AD pathology. Further longitudinal studies with larger sample sizes are necessary to evaluate the contribution of small vessel disease to cerebral Aβ accumulation.

## Supporting information

S1 Table Comparison of regional Aβ burden between low- and high-WML groups. (DOCX)

S1 Data Data underlying the findings. (XLSX)
