# Supplementary material for: Association between white matter lesions and cerebral Aβ burden
Source: PLoS One. 2018 Sep 24;13(9):e0204313. doi: 10.1371/journal.pone.0204313 (PMC6152974; doi:10.1371/journal.pone.0204313)
Supplement: S1 Table — (DOCX) [file pone.0204313.s001.docx]

**S1 Table. Comparison of regional Aβ burden between low- and high-WML groups**^*^

| **Group** | **Region** | **Side** | **WML burden**^†^ | | ***P value*** |
| --- | --- | --- | --- | --- | --- |
|  |  |  | **Low** | **High** |  |
| Overall | Frontal | Left | 1.27 (0.18) | 1.39 (0.32) | 0.032 |
|  |  | Right | 1.24 (0.18) | 1.38 (0.31) | 0.015 |
|  | Temporal | Left | 1.38 (0.19) | 1.48 (0.29) | 0.044 |
|  |  | Right | 1.30 (0.19) | 1.40 (0.29) | 0.055 |
|  | Parietal | Left | 1.34 (0.21) | 1.51 (0.38) | 0.013 |
|  |  | Right | 1.27 (0.22) | 1.41 (0.33) | 0.018 |
|  | Cingulate | Left | 1.53 (0.26) | 1.71 (0.35) | 0.010 |
|  |  | Right | 1.64 (0.21) | 1.76 (0.31) | 0.033 |
| CU^‡^ | Frontal | Left | 1.21 (0.12) | 1.19 (0.13) | 0.761 |
|  |  | Right | 1.19 (0.12) | 1.22 (0.25) | 0.681 |
|  | Temporal | Left | 1.30 (0.09) | 1.35 (0.20) | 0.456 |
|  |  | Right | 1.22 (0.10) | 1.21 (0.14) | 0.879 |
|  | Parietal | Left | 1.25 (0.15) | 1.23 (0.18) | 0.839 |
|  |  | Right | 1.19 (0.14) | 1.11 (0.12) | 0.300 |
|  | Cingulate | Left | 1.43 (0.16) | 1.64 (0.31) | 0.079 |
|  |  | Right | 1.57 (0.15) | 1.63 (0.31) | 0.556 |
| MCI[^§^](https://en.wikipedia.org/wiki/Section_sign) | Frontal | Left | 1.22 (0.17) | 1.40 (0.27) | 0.031 |
|  |  | Right | 1.19 (0.15) | 1.37 (0.23) | 0.015 |
|  | Temporal | Left | 1.36 (0.17) | 1.54 (0.29) | 0.043 |
|  |  | Right | 1.26 (0.16) | 1.41 (0.20) | 0.053 |
|  | Parietal | Left | 1.30 (0.18) | 1.55 (0.41) | 0.028 |
|  |  | Right | 1.21 (0.18) | 1.44 (0.35) | 0.024 |
|  | Cingulate | Left | 1.48 (0.23) | 1.74 (0.42) | 0.035 |
|  |  | Right | 1.62 (0.20) | 1.84 (0.31) | 0.029 |
| Dementia | Frontal | Left | 1.40 (0.19) | 1.43 (0.37) | 0.796 |
|  |  | Right | 1.38 (0.19) | 1.41 (0.35) | 0.762 |
|  | Temporal | Left | 1.49 (0.23) | 1.49 (0.30) | 0.993 |
|  |  | Right | 1.43 (0.23) | 1.44 (0.33) | 0.931 |
|  | Parietal | Left | 1.49 (0.24) | 1.55 (0.39) | 0.633 |
|  |  | Right | 1.42 (0.25) | 1.46 (0.33) | 0.694 |
|  | Cingulate | Left | 1.70 (0.30) | 1.71 (0.35) | 0.950 |
|  |  | Right | 1.74 (0.23) | 1.75 (0.32) | 0.879 |

^*^All values are mean regional SUVR (SD). ^†^WML: white matter lesion; ^‡^CU: cognitively unimpaired; [^§^](https://en.wikipedia.org/wiki/Section_sign)MCI: mild cognitive impairment
